# Measures of odor and lateralization thresholds of acrolein, crotonaldehyde, and hexanal using a novel vapor delivery technique

**DOI:** 10.1371/journal.pone.0185479

**Published:** 2017-09-26

**Authors:** Lena Ernstgård, Aishwarya M. Dwivedi, Johan N. Lundström, Gunnar Johanson

**Affiliations:** 1 Work Environment Toxicology, Institute of Environmental Medicine, Karolinska Institutet, Stockholm, Sweden; 2 Division of Psychology, Department of Clinical Neuroscience, Karolinska Institutet, Stockholm, Sweden; Duke University, UNITED STATES

## Abstract

**Introduction:**

Humans are exposed to aldehydes in a variety of environmental situations. Aldehydes generally have a strong odor and are highly irritating to the mucous membranes. Knowledge about odor perception and especially irritation potency in humans is thus essential in risk assessment and regulation, e.g. setting occupational exposure limits. However, data on odor and irritation are lacking or limited for several aldehydes. The aim of the study was to determine the odor and lateralization thresholds of some commonly occurring aldehydes. Acrolein and crotonaldehyde where chosen as they are formed when organic material is heated or burned, e.g. during cigarette smoking. n-Hexanal was also included as it is emitted from wood pellets and fibreboard.

**Material and methods:**

To study odor and lateralization thresholds of these aldehydes, a novel, inexpensive olfactometer was designed to enable delivery of reliable and stable test concentrations and thus valid measures of thresholds. The delivery system consists of seven syringe pumps, each connected to a Tedlar bag containing a predefined concentration of the tested aldehyde vapor. To validate the threshold measures, a test-retest was performed with a separate method, namely odor delivery via amber bottles. Twenty healthy naïve individuals were tested.

**Results:**

The median odor thresholds of acrolein, crotonaldehyde and hexanal were 17, 0.8, and 97 ppb, respectively. No lateralization threshold could be identified for acrolein (highest tested concentration was 2 940 ppb in 5 subjects), whereas the medians were 3 and 390 ppb for the latter two. In addition, odor thresholds for n-hexanal were also determined using two methods where similar results were obtained, suggesting that the olfactometer presentation method is valid.

**Conclusion:**

We found olfactory detection and lateralization thresholds (except for acrolein) in alliance with, or lower than, previously reported in naïve subjects. The new olfactometer allows better control of presentations timing and vapor concentration.

## Introduction

Many aldehydes are formed not only during combustion, but also as a result of chemical and microbiological processes in organic materials, such as e.g. fiber board, wood based panels, wood pellets, polyethylene resins, composting and swine stables [1,2,3,4,5,6]. Thus, humans are exposed to airborne aldehydes in a variety of environmental situations. The aldehydes generally have a strong odor and are highly irritating to the mucous membranes. The hazards of aldehyde exposure, as of any airborne chemicals, cannot be evaluated by the quality or intensity of the odor, since odor correlates poorly with toxicity. However, the odor can be used in a recognition phase to identify the nature of the exposure. Aldehydes, especially the short-chained ones, are strong irritants to the mucous membranes of the airways. Knowledge about odor perception and irritation potency in humans is thus essential in risk assessment and regulation, e.g. when setting occupational exposure limits. However, data on odor and irritation are lacking or limited for several aldehydes.

Acrolein (2-propenal) is a reactive aldehyde formed when heating or burning organic matter, such as wood burning, tobacco smoking, cooking and diesel fuel combustion. A large proportion of the population is predominantly exposed via air (smoke from cigarettes, automobiles, industrial processes and structural and vegetation fires). Acrolein has been described to have an acrid, pungent odor with sensory irritating effects [7]. Weber-Tschopp et al. [8] performed a study where subjective methods (questionnaires), eye blink frequency and respiration rate were used to examine the irritation of acrolein. They found eye, nasal and throat irritation beginning at 90, 260 and 430 ppb, respectively. Nagata [9], using a trained panel and the triangle bag method, reported an odor threshold (OT) of 3 ppb.

Crotonaldehyde (2-butenal) occurs widely in nature. It is highly reactive and used in the synthesis of crotonic acid (2-butenoic acid), the food preservative sorbic acid (E 200) and many other chemicals. Crotonaldehyde is one of the more potent irritants among the alfa, beta-unsaturated aldehydes, and appears to be slightly more irritating than acrolein and formaldehyde [10]. The OT has been reported to 160 ppb [11] and 23 ppb [9]. Trofimov [12] reported a threshold for mucous membrane irritation of 170 ppb for humans.

Hexanal (caproaldehyde) occurs naturally in plants and animals and has been identified in approximately 100 food items [13]. Hexanal is emitted from wood and more so from processed wood products and has been identified as a major component in emissions from wood pellets. The odor of hexanal is described as “grassy”. Mild irritation was reported after a two hours of whole-body exposure to 10 ppm of hexanal [14]. Li Zheng [15] reported odor and sensory irritation thresholds of 25 and 281 ppb, respectively.

As illustrated above, previous studies have reported a wide range of odor detection and lateralization thresholds. The key problem is likely that different methods have been used and that the true concentration presented to the test person may differ to varying extents from the nominal/theoretical concentration. The most common method to measure the OT is to present the stimuli in various types of bottles. This is a simple and inexpensive method, however, while the concentration in the aqueous phase may be controlled and kept constant, the concentration in the headspace that is actually presented to the participant is far more difficult to control and may vary widely depending on e.g. temperature, liquid and head-space volumes, and shape and size of bottle, bottleneck and bottle mouth. There are only a few olfactometers available on the market that is able to present vapors at well-defined concentrations. However, most if not all are expensive and/or are technically demanding. Thus, development of an easy to handle and yet reliable instrument to present reliable concentrations of chemical to the test subject is needed.

Chemical substances typically elicit odor sensations at relatively low concentrations followed by sensory irritation at higher concentrations. It has proved difficult to specify the lateralization threshold in normosmic subjects, as the odor sensations interfere with the perception of irritation. This is presumably because of the subjective ambiguity regarding the points at which the sensation of the odor itself becomes an irritation and the sensation takes on an irritation character via stimulation of the trigeminal nerve, i.e. where the line is drawn between the psychological and physiological irritation [16]. Subjective reporting of irritation is therefore unreliable. A more objective measure is to assess the lateralization threshold (LT); a measure based on our inability to correctly identify which nostril received an odor if the contralateral nostril receives and equal amount of air [17,18]. Only if the odor activates the trigeminal nerve to some degree and provoke a sensation of irritation are we able to correctly lateralize the nostril receiving the stimuli. Thus, the ability to perform accurate lateralization judgments above chance is a valid measure of a nasal sensory irritation threshold.

The aim of the present study was to determine the odor and lateralization thresholds for the three aldehydes; acrolein, crotonaldehyde, and hexanal. Determination of odor thresholds may serve as a cue of “bad-smell” which could serve as a first-line warning system. Measurement of lateralization threshold is an objective measure of sensory irritation in the nose and the endpoint is important in setting occupational exposure limits. To that purpose a novel technique was developed in order to generate, measure, maintain and present low, yet stable, concentrations of chemical vapor.

## Material and methods

### Subjects

Twenty healthy volunteers (average age 28, range 22–40 years, 7 men) were recruited by advertisement at Karolinska Institutet, Stockholm University, and the Royal Institute of Technology. To ensure that the subjects were healthy and had a normal sense of smell they underwent a small medical examination prior to start of the study, including anosmia screening with Sniffing Sticks (Burgharts’s Sniffing Sticks identification test). Anosmics, smokers, snuff users and subjects with a history of allergy or other chronic diseases were excluded. In addition, females performed a pregnancy test (hCG 25 IU/L ColibriCheck, Colibri Medical AB, Sweden) before all three individual exposure sessions to avoid unnecessary fetal exposure. The volunteers were informed about the design of the study, possible hazards, and their right to immediately and unconditionally interrupt the study. Each participant signed a written informed consent and the study was approved by the Regional Ethical Review Board in Stockholm (dnr 2015/10-31).

The participants entered the testing room at least 10 min before start of the experiment as to be preconditioned to the environment.

### Olfactometer

**Fig 1:**
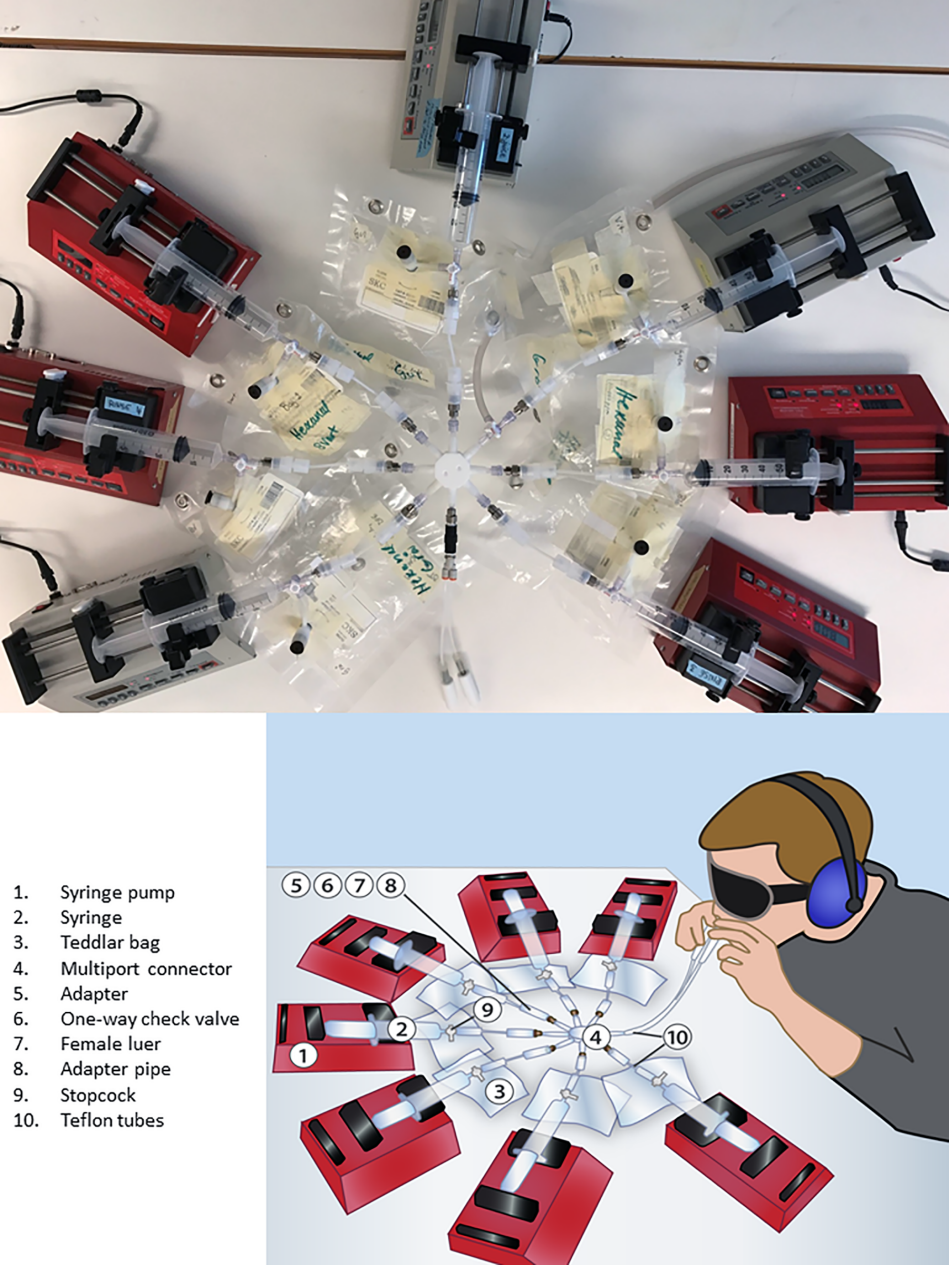
Photo (A) and conceptual drawing (B) of the olfactometer and test setup, with the seven pumps connecting Tedlar bags with different concentrations of aldehyde vapor to an 8-way mixing valve. The eight port is connected to a birhinal nose piece. For parts also see Table 1.

A novel, inexpensive olfactometer was constructed to enable stable and reliable generation of low concentrations of test vapor (Fig 1A and 1B). The delivery system consists of seven syringes, each one mounted in a syringe pump and connected with tubing via a three-way valve to a Tedlar bag for supply of test vapor and to an eight-way mixing valve (multiport connector, Cole-Parmer) for delivery of vapor. Six of the Tedlar bags (SKC, Eighty Four, PA) contain different predefined concentrations of the test chemical and the seventh bag contains clean air. The flow rate of each pump (BS 9000, Braintree Scientific, Denmark) is set to 23 ml/min. Each bag is also equipped with a one-way check-valve (Cole-Parmer) to prevent flow direction problems. The eighth outlet of the mixing valve connects to a birhinal nose piece via a one-way check-valve, a splitter and tubes. The nose pieces were made in house from tetrafluoroethylene (PTFE) and anatomically shaped so as to fit well with the nostrils. The tubes are kept short (10 cm) to minimize the dead space from the eight-way valve to the nose pieces.

**Table 1:** Parts list.

Parts	Supplier	Part number	Number in Fig 1
Syringe pump, BS 9000	Braintree Scientific		1
Syringes, plastic, Luer-Lok 60 ml	Becton Dickinson	300865	2
Teddlar bags	SKC	Depending on size	3
Multiport connector, PFTE	Cole Parmer	06473–05	4
Adapter with caps, PFTE cons	Cole Parmer	06471–81	5
Luer, one-way check-valves, DEHP-with silicone diaphragms	Cole Parmer	30505–92	6
Female luer x 1/8”NPT	Cole Parmer	41507–86	7
Adapter pipe F 1/8 x 1/8”, PFA	Cole Parmer	31320–50	8
Stopcock 4-way male lock, polycarbonate	Cole Parmer	30600–04	9
Teflon tubes, 1/16” id x 1/8” od	Cole Parmer	6605–27	10

**Fig 2:**
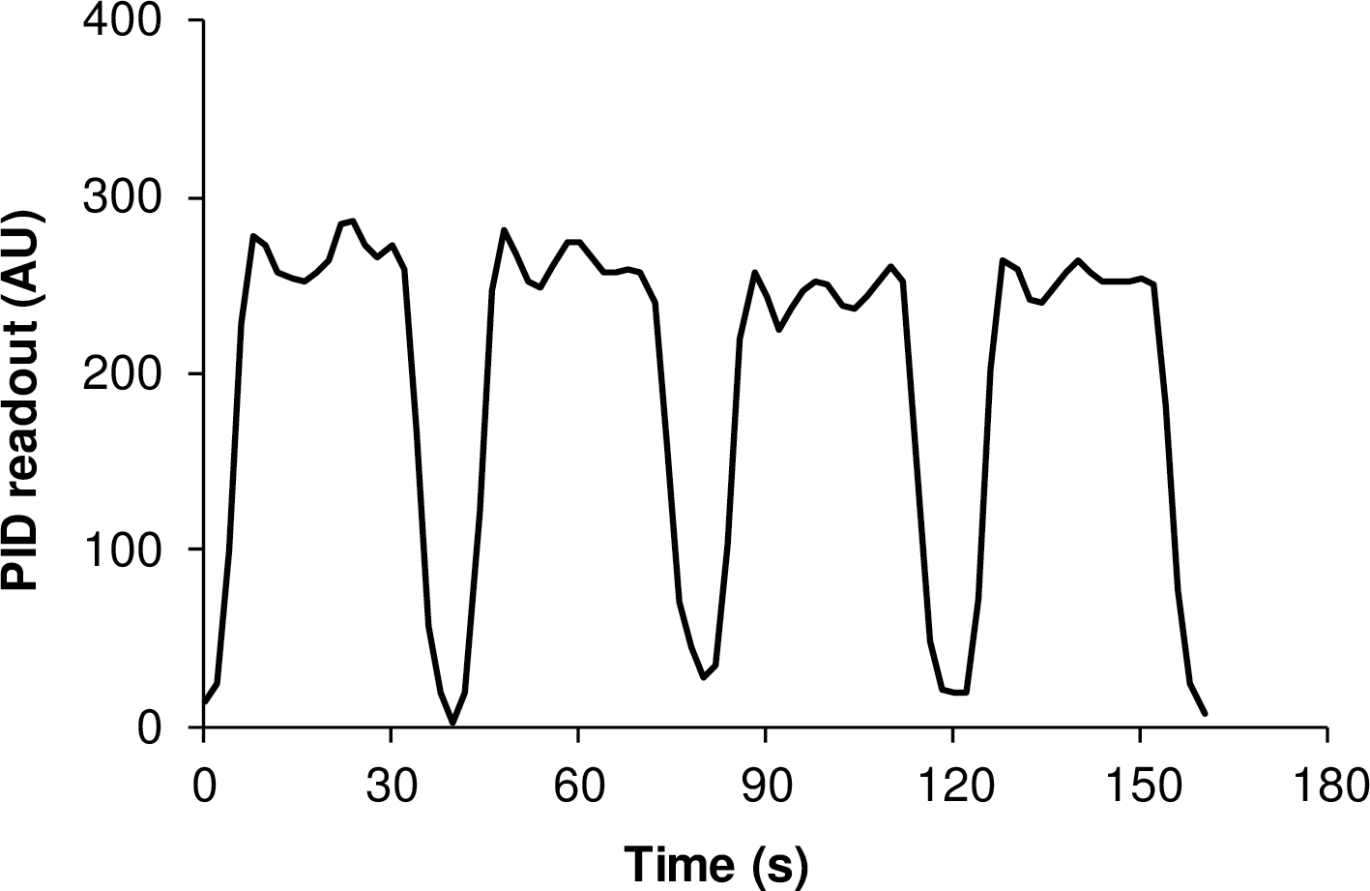
Photoionization detector (PID) readout over time measured at the tip of the nose piece. The variation of four consequent presentations of 4000 ppb n-hexanal from a syringe is shown. AU = arbitrary units.

A dilution of aldehyde vapor of known concentration was generated by injection of an appropriate amount of test chemical into a Tedlar bag (starting bag) containing a known volume of clean air by means of a calibrated pump (AirCheck sampler, Model 224-PCXR8, SKC, Inc., Eighty Four, PA). Complete vaporization was assured by gentle heating of the bag from the outside with a hair dryer. The six test concentrations were then obtained by adding different volumes from the starting bag to six new Tedlar bags with known volumes of clean air. One bag contains clean air only. Thus, six different test concentrations and clean air could easily be tested in series by shifting the position of the eight-way valve. New bags were prepared every morning and calibrated by gas chromatography (GC). The gas chromatograph (Auto system, Perkin Elmer, Norwalk, CT) was equipped with a 2-ml sample loop, a wide bore capillary column (CP wax, Chrompack no 7658, 25 m, 0.53 ID, 2.0 mm) and a flame ionization detector. Nitrogen was used as a carrier gas at a column pressure of 9.6 psi (2.5 ml/min). The temperature of the injector was 250 C and of the detector 275 C. The column temperature was kept at 60 C. The retention times for acrolein, crotonaldehyde, and hexanal were 0.95, 2.3, and 2.8 min, respectively. The total analysis time was 3.5 min. The limit of detection (LOD), calculated from the minimum peak area that could be identified by the integrator, was about 0.01 ppm for all three aldehydes. The GC method and preparation of standards were as previously described by [19]. The stability and reproducibility of the delivered concentration of aldehyde were checked by means of a photo-ionization detector (PID, ppb RAE, model PGM-7240, RAE Systems, California, USA) (Fig 2).

### Test substances

Acrolein 99% (FLUKA Analytical, Sigma-Aldrich, Buchs Switzerland), crotonaldehyde 99.5% (Sigma-Aldrich, Buchs Switzerland) and hexanal 98% (Merck, Darmstadt, Germany) were used in the odor tests.

### Odor threshold

In the olfactometry tests, air from bags with different concentrations of aldehyde vapor was pumped through via the eight-way valve to the birhinal nose piece. The subject started to inhale 20 s after turning on the pump (set to time with vapor arrival at the nose) and inhaled for 5 s where after the pump was turned off. After each presentation, high-flow pressurized clean air is pumped through the system for 10 s to purge residual odors. After another 5 s, the trial procedure was repeated (40 s test cycle). The subject was alternately presented to aldehyde vapor and clean air and had to decide when he or she was presented with aldehyde. After each choice, the subject also rated how confident he or she was that the decision was correct using a verbal scale ranging between 50 (no confidence at all) 100% (very high confidence). The subject was blinded to all operations by the investigator by wearing an eye mask.

The aldehydes were presented to the subject at stepwise increasing concentrations until the odor was perceived (Table 2). The odor threshold was defined as the first concentration at which the subject was able to correctly distinguish between aldehyde and clean air three times in a row. If incorrect, the concentration was increased one step. The stimuli were presented in a pseudo randomized predefined order. A pilot study was performed in eight subjects prior to the main study, to determine the concentration ranges.

**Table 2:** Measured exposure concentrations of the selected aldehydes (mean ± S.D). Levels in bags 1–5 of acrolein and crotonaldehyde and 1–4 of hexanal were calculated from the measured concentrations.

	Acrolein (ppb)	Crotonaldehyde (ppb)	Hexanal (ppb)	
Level	Bags	Bags	Bags	Bottles
1	2.7	0.8	12	12
2	5.4	2.2	39	39
3	10.7	5.3	117	117
4	22.2	13.2	390	308
5	44.5	35.2	1414 ±207 (n = 10)^#^	1363±193 (n = 10)^#^
6	88.5 ±7 (n = 10)^#^	92.5 ±17 (n = 11)^#^	3911±252 (n = 10)^#^	4699 ±792 (n = 10)^#^

^#^ Measured concentrations by gas chromatography.

No arrangements for fugitive gases were made as the released volumes were minimal, resulting in at least million-fold dilution in the testing room.

### Lateralization threshold

We also assessed the lateralization threshold (LT) of the three aldehydes. An additional pump supplying clean air was added to the delivery system allowing the nostrils to be separately and interchangeably exposed to an equal flow of clean air and air with test chemical. The subject was presented with clean air in one nostril and aldehyde vapor in the other up to 9 times per concentration step, with the direction (left or right nostril) of presentation being varied in a predefined random order. The LT was defined as the concentration at which the subject correctly identified which nostril is exposed to test chemical in at least 7 of the 9 trials. After each trial the subject also rated the confidence in the decision on the 50–100% scale. The lateralization test started at the previously determined individual OT concentration.

### Odor threshold by amber bottles

To verify the results from the novel equipment, the OT of hexanal was also assessed using amber bottles. Ten of the previous subjects were tested by sniffing air from 60 ml glass bottles containing 10 ml of aqueous solutions hexanal. The procedure was the same as in the olfactometry tests, i.e. the bottles were presented pairwise (clean air and hexanal) for 5 s with 30 s between the presentations in the same predefined order. As in the olfactometry, the subject had to decide which bottle contained hexanal and rate how confident he was. The solutions were prepared so that the concentrations in the head space of the bottles would correspond to those used in the olfactomtery tests (Table 2). The concentrations were confirmed by GC analyses of the two highest concentrations. Samples of head-space vapor from the bottles were taken by a 10 ml syringe and injected directly to the sample loop of the GC. The GC method and preparation of standards were as previously described by Dwivedi et al. [19].

### Calculations and statistical analyses

The concentrations of the bags were corrected for each individual to the daily measurement. Confidence ratings were grouped by 10% intervals for all three aldehydes and levels as well as OT and LT tests and correlated to the proportion correct choices (S1 File).

## Results

### Equipment

Initial in-line measurement with the PID showed that the delivery system responded rapidly and reliably to a shift in valve position. The air passing the nose tip reached 90% of the concentration in the bag within 15 seconds (Fig 2).

### Odor threshold

**Fig 3:**
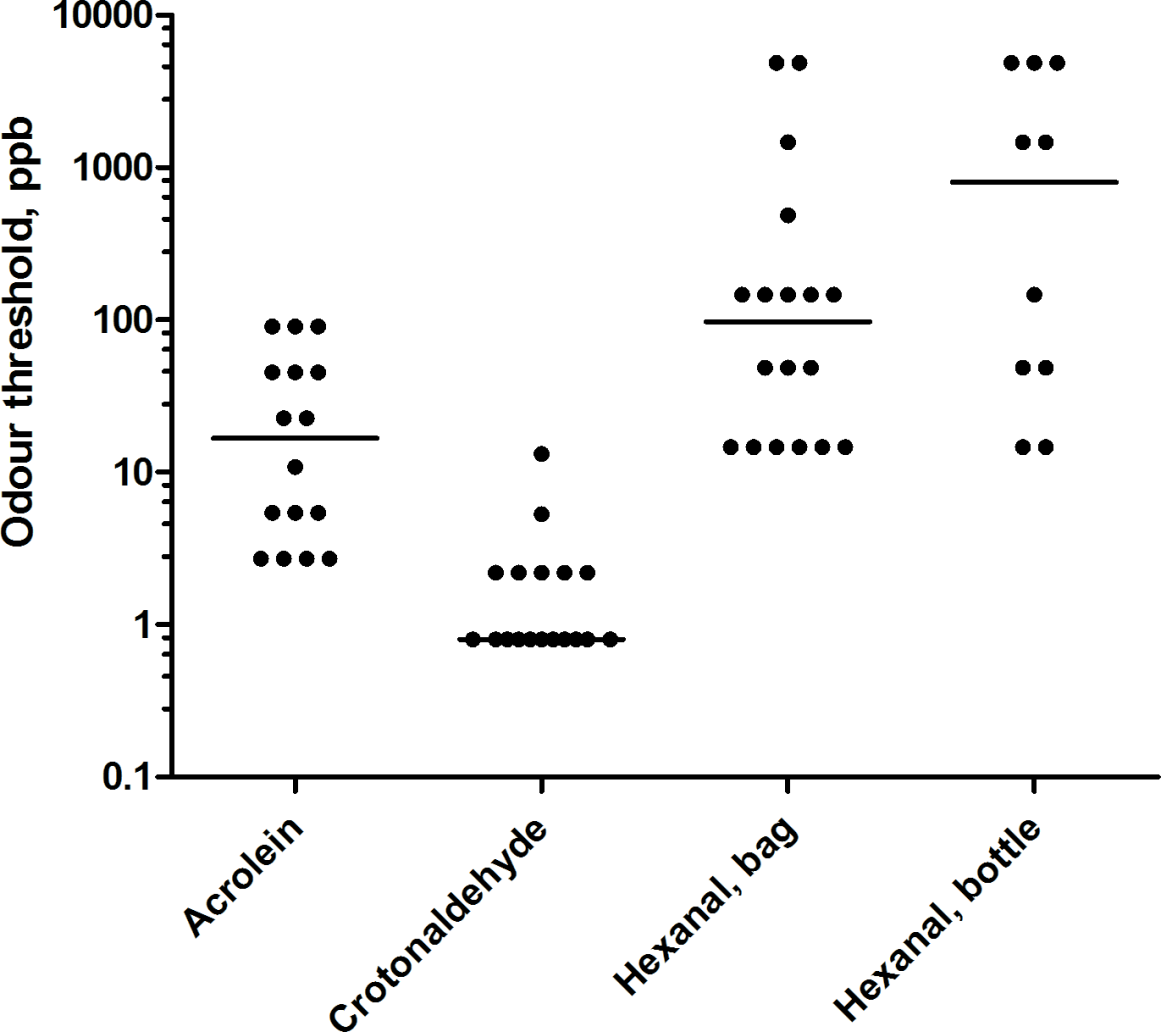
Measured odor thresholds of acrolein, crotonaldehyde, and hexanal. Each dot represents the threshold for one individual. The horizontal lines represent median.

The individual OT data are presented in Fig 3 (S 1 File). The median OT for acrolein was 17 ppb ranging from the lowest to the highest (2.7–88.5 ppb, Table 2) concentration presented. Two of 18 subjects did not detect any odor even at the highest test concentration. Eleven of 20 subjects detected the odor of crotonaldehyde already at the lowest concentration presented (i.e. the median 0.8 ppb). On the other hand, two subjects did not perceive any odor even at the highest concentration (92.5 ppb). The median OT for hexanal was 97 ppb, individual values ranging from the lowest to the highest concentration presented (11.7–3911 ppb). Again, two subjects did not perceive any odor even at the highest concentration of hexanal.

To test the validity of the olfactometer, the OTs of hexanal were also determined with amber bottles in 10 subjects six months after the olfactometry tests. The delay was deliberate to prevent potential carry-over effect due to familiarity and repeated exposure. The bottle test gave similar OTs as the olfactometry (Spearman rho = 0.46).(Fig 3).

### Lateralization threshold, LT

**Fig 4:**
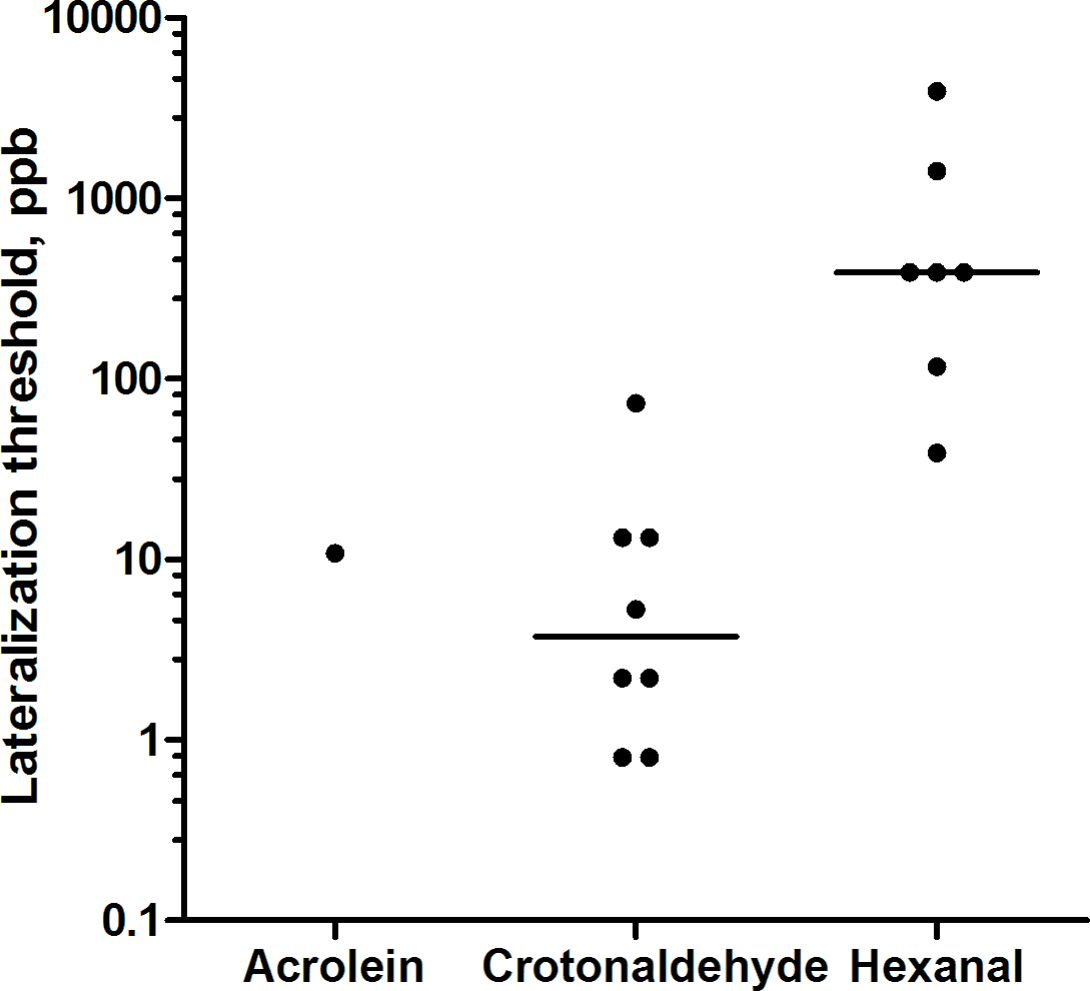
Measured lateralization thresholds of acrolein, crotonaldehyde, and hexanal. Each dot represents the threshold for one individual. The horizontal lines represent median.

The individual results on LT are presented in Fig 4 (S 1 File). An LT for acrolein could only be obtained in one of the 20 subjects. This one subject determined an LT at 10.7 ppb. For crotonaldehyde, LTs were identified in 8 subjects with a median value of 3 ppb. The variability in LT between the subjects was hundred-fold and covered the entire concentration range tested (0.8–92.5 ppb, Table 2). The median LT for hexanal was considerably higher, 390 ppb, and was identified in 7 subjects, again with a hundredfold range from 39 to 3911 ppb.

Five of the subjects that failed to identify an LT for acrolein or hexanal were tested again at later time points with higher concentration ranges (acrolein 90 ppb– 2940 ppb, hexanal 5 000–78 000 ppb). The tested subjects were still unable to identify an LT.

**Fig 5:**
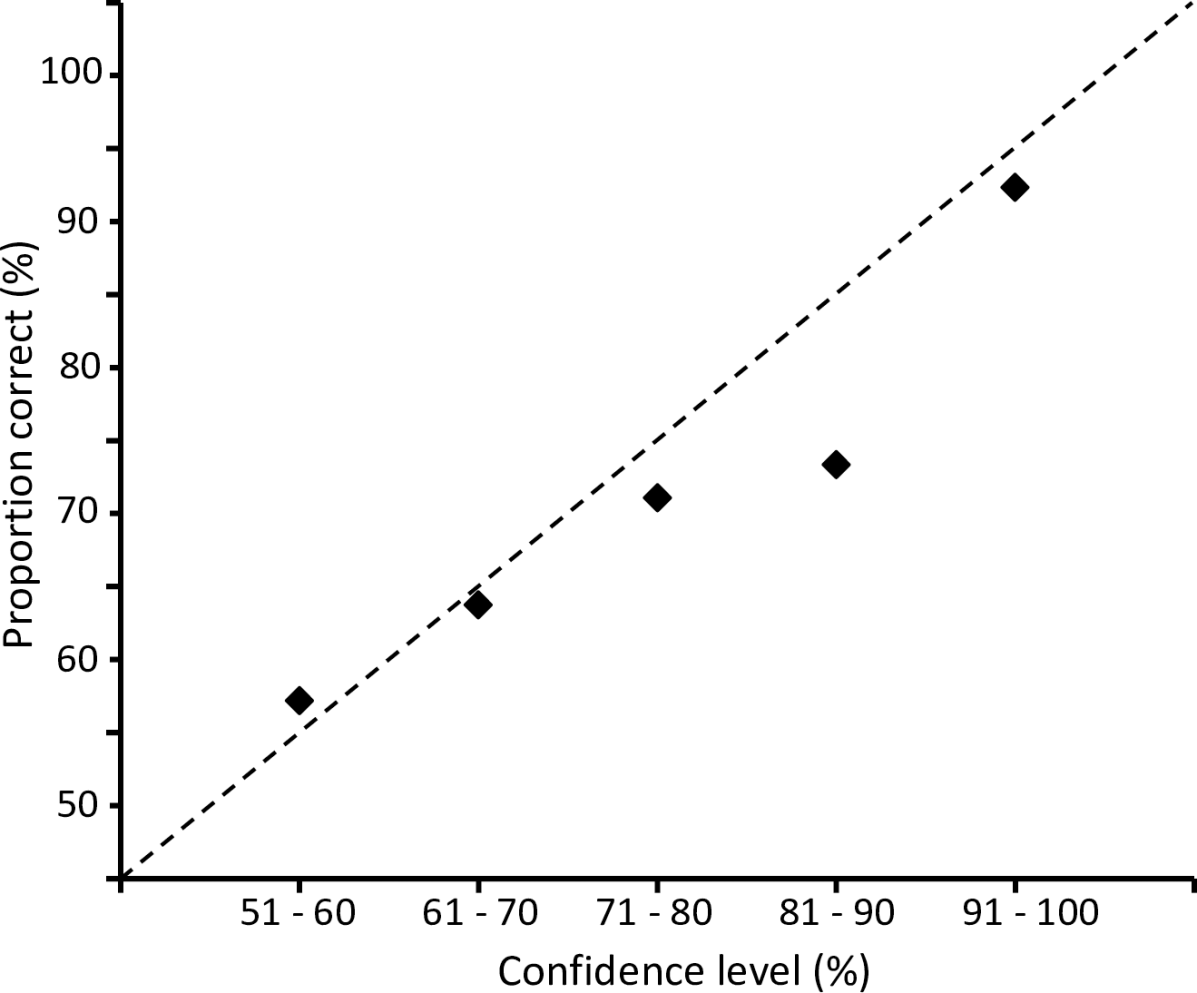
Correlation between all confidence ratings and proportion correct choices (%). Ratings were averaged in 10%-intervals.

Visual inspection of the confidence ratings showed a good fit with the proportion correct choices, thus indicating that participants were well calibrated in their confidence assessments (Fig 5). A subsequent weighted least square analyses gave that there was no significant difference between the observed values and the line of perfect calibration (*t* = .09, *p* = .89).

## Discussion

To assure presentation of precise vapor presentation, a novel simple olfactometer was developed. The results of the exposure to vapors of three aldehydes show that this olfactometer produces valid results among volunteers in terms of OT and LT values. The time between the presentations is less than 1 min depending on the air flow rate. A higher air flow would give an even shorter test cycle, as the desired concentration is reached faster. However, a higher flow also increases the risk of drying out the respiratory mucosa resulting in a pain sensation that hinders reliable measurements [20]. It has been suggested that longer inter-stimulus presentation intervals are required in experiments with odorous irritants, like aldehydes, compared with pure odorants [21]. Moreover, previous data has demonstrated that odor stimuli presented beyond the often used 1–3 s presentation time are detected at lower concentrations. Whether this is due to an increased trigeminal sensation or simply due to a higher ecological relevance is not known. However, it could be speculated that the olfactory system is not primarily designed to detect and process short odor stimuli but rather, much like our other senses, given the higher prevalence in our everyday life, optimal detection and processing is accomplished by longer odor exposures. Thus, the extended presentation of 5 s used in the present setup might be advantageous from a stimulus detection perspective.

The results of the amber bottle test provide additional support that the stimulus presentation method is valid. Thus, all ten subjects had an OT in the same range as with the bag method (Fig 3). Smeets et al [22] states that very few findings have been published with regard to the test–retest reliability of the OT assessment procedure and that the expected individual thresholds may vary over time due to natural fluctuations. Taking into consideration the six months that elapsed between the olfactometry and amber bottle test, the possibility of natural fluctuation and the low number of subjects in the bottle test (n = 10), the test-retest reliability seems acceptable (Spearman rho = 0.46).

Although there is good general agreement, our results show some discrepancies compared to previously reported OT and LT values, obtained with different methodology. Thus, we obtained a higher OT for acrolein (17 ppb) than reported by Nagata [9], 3 ppb, but lower than that reported by Amoore and Hautala [11], 160 ppb. Further, we found a lower OT for crotonaldehyde (0.8 ppb, the lowest tested concentration) than previously reported value of 120 ppb by Amoore and Hautala [11] and also lower than the 23 ppb reported by Nagata [9]. Nagata [9] used a trained panel, yet our OT obtained with naïve subjects was even lower. Similar discrepancies are present for hexanal where our OT of 97 ppb is higher than those reported by Zheng [15] of 25 ppb, using the P_50_ method (50% probability of detection), and by Hall and Andersson [23] of 11 ppb using a method of constant stimuli. In view of the seemingly large intra-individual variability in OTs, the apparent discrepancies between studies are not surprising.

In 17 of 18 subjects, no LT was found for acrolein up to 90 ppb. In additional testing with 5 subjects no LT was found even up to 2940 ppb. We have previously performed whole body chamber exposures to acrolein, starting with a pilot study where 8 subjects were exposed to increasing concentrations (0, 20, 40, 70, 100, 200, and 300 ppb, 10 min per level). No clear effects were seen on the ratings of nasal irritation. However, one subject rated marked irritation already at 40 ppb, while the other seven rated no or little irritation at all levels. In the main study, eighteen volunteers were exposed for 2 h to 0, 50 and 100 ppb acrolein. Again, no significant increase in the rating of nasal irritation was seen. In contrast, a significant increase in eye irritation was seen, with ratings increasing over time and with exposure level [19]. Considering the differences in exposure setup (nose only vs whole body), exposure duration (5 s vs 2 h) and endpoint measurement (categorical vs magnitude rating), the results from the present lateralization study and our previous chamber study seem to be in reasonable agreement. Furthermore, both studies indicate a large inter-individual variability in sensitivity to acrolein. Several studies indicate that acrolein causes irritation via the sensory irritant receptor, Transient Receptor Potential Ankyrin 1 (TRPA1). For example, TRPA1-deficient mice show a loss of sensitivity to acrolein and other electrophilic substances [24,25,26]. Acrolein probably activates TRPA1 receptor via covalent binding to the TRPA1 protein that opens the cation conducting channel. This leads to neuronal depolarization and calcium ion influx into the sensory nerve endings, resulting in sensory irritation [27]. Considering the mechanism of covalent binding to TRPA1, modification of the TRPA1 protein and the resulting irritation will build up gradually over time. Thus, in view of short exposure duration in our LT test (5 s per exposure level), it may be that too low concentrations of acrolein were used. We obtained a median LT value for crotonaldehyde of 3 ppb among 8 subjects whereas no LT could be identified in the remaining 12. For comparison, Trofimov [12] reported a threshold for mucous membrane irritation of 170 ppb. The median LT of hexanal was 390 ppb found in 6 of 20 subjects which are in agreement with a study by Li Zheng [15] who reported sensory irritation at 281 ppb (measured by ratings). We have previously also performed whole body chamber exposures to hexanal (0, 2 000 and 10 000 ppb for 2 h). A significant increase in ratings of nasal irritation was seen already at 2 000 ppb, beginning at after 60 min [14]. Considering the differences in exposure setup (nose only vs whole body), exposure duration (5 s vs 2 h) and endpoint measurement (categorical vs magnitude rating) rated irritation, the two results are in reasonable agreement. One may speculate that with a broader concentration range an LT could have been identified in more subjects. Five of the subjects that failed to find an LT were tested with concentrations up to 78 000 ppb of hexanal later, still they could not identify any LTs. Thus, the individual variation in trigeminal intranasal sensitivity seems to be large for aldehydes.

The study has a few limitations that might impact the interpretation of the obtained result. The same size is not excessive and given the inability to determine a valid LT for acrolein, it would be have been advantageous if a larger a prio sample size had been set. A significant larger sample size would have allowed analyses of potential demographical factors mediating the results as well as allowed us more detailed analyses of individual differences, a parameter known to influence odor detection measures [28], and their potential mechanisms. Moreover, we did not control, nor regulate, participants breathing; either during odor delivery or throughout the experiment. Regulating breathing is experimentally difficult due to large variability in preferred breathing rate and longer periods of mismatch between allowed and preferred rate is viewed as very aversive. That said, participants were instructed to breathe through their nose during the odor delivery phase but it is conceivable that mouth breathing did occurred despite this instruction. This might have negatively affected the reliability of the obtained measures. Future studies should aim to include a much larger sample and actively measure compliance with breathing instructions.

Overall, the wide variation in threshold definition, sample presentation, panel selection and data interpretation makes it difficult to compare OT and LT values between studies. Nevertheless, our study suggests that better control of presentations timing and vapor concentration results in a tendency towards lower OT and LT values. However, additional studies need to be performed to evaluate the reliability and validity of the novel olfactometer.

## Conclusion

In the present study, we determined olfactory detection thresholds for vapors of acrolein, crotonaldehyde, and hexanal that where either in alliance with or lower than previously reported in naïve subjects. Regarding irritation, lateralization thresholds were established for crotonaldehyde and hexanal and found to be lower (crotonaldehyde) or similar to previously reported values. Moreover, we have successfully constructed a novel inexpensive olfactometer which enables stable and reliable presentation of chemical vapors and thereby valid and reliable measures of odor and lateralization threshold. The thresholds presented herein could be a valuable source of information when developing exposure standards such as occupational exposure limits and guidelines for indoor environment.

## Supporting information

S1 File Data on individual odor threshholds (OT), lateralization threshold (LT) and confidence rating data. (XLSX)
